# Clinical Neuropathology Practice News 2-2013: immunohistochemistry pins IDH in glioma – molecular testing procedures under scrutiny 

**DOI:** 10.5414/NP300622

**Published:** 2013-02-28

**Authors:** Matthias Preusser, Martin van den Bent

**Affiliations:** 1 Department of Medicine I and Comprehensive Cancer Center CNS Unit, Medical1397645907University of Vienna, Austria,; 2 Department of Neurology/Neuro-Oncology ErasmusMC - Cancer Institute, Rotterdam, The Netherlands

**Keywords:** isocitrate dehydrogenase, glioma, immunohistochemistry, sequencing

## Abstract

Isocitrate dehydrogenase 1 (IDH1) gene mutations occur in ~ 60 – 90% of diffuse and anaplastic gliomas and secondary glioblastomas. IDH status is strongly associated with patient survival times and IDH testing is relevant for clinical patient management and for stratification in clinical trials. A recent interlaboratory ring trial shows that immunohistochemistry is a highly reliable method to detect the most common IDH mutation (R132H), while IDH gene sequencing is less robust. These results support initial immunohistochemistry and subsequent gene sequencing in cases with negative or inconclusive immunostaining result as valid algorithm for IDH testing. Furthermore, they highlight the need for strict quality control of DNA-based biomarker analyses on formalin-fixed and paraffin-embedded tumor samples.

## IDH testing under scrutiny 

Isocitrate dehydrogenase 1 (IDH1) gene mutations are found in 60 – 90% of diffuse and anaplastic gliomas and secondary glioblastomas [1, 2]. IDH testing supports neuropathological differential diagnosis, e.g., for differentiation of oligodendrogliomas from other tumors with clear cell appearance or of glioma from reactive gliosis [3, 4]. Furthermore, IDH mutations confer a favorable survival prognosis and appear relevant as stratification factor in clinical trials on glioma [5]. Assessment of the IDH status may be performed by DNA-based methods or by immunohistochemical detection of the mutated protein [6, 7, 8]. A recent study assessed the reliability of these IDH test methods on routine formalin-fixed and paraffin-embedded tumor tissue samples in an interlaboratory ring trial involving 6 international neuropathology laboratories [9]. Immunohistochemistry was found to be highly reproducible despite the fact that the staining protocols varied between the laboratories. In contrast, IDH sequencing procedures yielded discordant results in 2 of 6 laboratories. These results support a previously proposed algorithm for IDH testing based on initial anti-IDH1-R132H immunohistochemistry and subsequent gene sequencing in cases with negative or inconclusive immunostaining results [7]. Importantly, gene sequencing procedures need to be strictly quality controlled. 
